# Predictors of PTSD Symptom Reduction in a Secondary Analysis of a Randomized Controlled Clinical Trial

**DOI:** 10.3390/jcm15010090

**Published:** 2025-12-23

**Authors:** Tyler C. Smith, Besa Smith, An-Fu Hsiao, Andrea Munoz, Chelsea Aden, Jennifer Lai-Trzebiatowski, Megan Jung, Trevor J. Murphy, Michael Hollifield

**Affiliations:** 1 Department of Health Services and Leadership, School of Health Professions, National University, San Diego, CA 92123, USA; 2 Analydata, 3835 Centraloma Drive, San Diego, CA 92107, USA; 3 Tibor Rubin VA Medical Center, 5901 E. 7th St, Long Beach, CA 90822, USAandrea.munoz@va.gov (A.M.); trevor.murphy@va.gov (T.J.M.);; 4 Department of Medicine, Health Policy Research Institute and General Internal Medicine, University of California Irvine, 100 Theory, Suite 110, Irvine, CA 92697, USA; 5 Department of Psychiatry and Behavioral Sciences, George Washington School of Medicine & Health Sciences, Washington, DC 20037, USA

**Keywords:** post-traumatic stress disorder, PTSD, acupuncture, clinical trial, predictors

## Abstract

**Objective:** In a secondary analysis of a randomized, sham-controlled trial, we prospectively investigated baseline comorbidities, demographics, and intervention as predictors of clinically meaningful (≥15-point) CAPS-5 reduction in PTSD symptom reduction. **Methods:** This four-year (2018–2022), two-arm, parallel-group, prospective randomized placebo controlled clinical trial was conducted at the Long Beach VA Healthcare System among 71 treatment-seeking 18–55-year-old Veterans with chronic combat-related PTSD. Hierarchical and backward multivariable logistic regression models were conducted to compare the predictive capabilities of discriminating between 15-point reduction or more in CAPS-5 at follow-up. **Results:** Hierarchical multivariable logistic modeling found demographic variables alone provided a nearly acceptable prediction of 15-point reduction (c-statistic = 0.69) while clinical assessments alone provided an acceptable prediction (c-statistic = 0.75). Together, the baseline demographic and clinical variables indicated strong prediction (c-statistic = 0.92) and the addition of the group intervention variable increased the prediction (c-statistic = 0.94). In a backwards stepwise regression retaining variables with an alpha = 0.10 significance, females (adjusted odds ratio (AOR) = 14.7), and those receiving acupuncture (AOR = 4.17), indicating better physical health (AOR = 1.14) and less pain (AOR = 0.95), were statistically more likely to result in a 15-point CAPS-5 reduction at follow-up after controlling for other variables in the model. **Conclusions:** In this small sample, demographic and baseline clinical variables were independently predictive of symptom reduction and, together with the acupuncture intervention, presented a near perfect prediction of PTSD symptom reduction, though further validation is warranted. Patient characteristics that may indicate a more favorable response for PTSD symptom reduction include less baseline pain, better physical functioning, females, increasing age, and sociodemographic variables including higher income and not employed.

## 1. Introduction

For two decades, the US military was engaged in combat-intensive deployments in Iraq, Afghanistan, Syria, and neighboring countries initially called the Global War on Terror (GWOT). These operations came to a close in August 2021 with the withdrawal of US forces from Afghanistan after over 7000 US service members had lost their lives and an additional 50,000 were wounded in action [1]. The lengthy combat engagements resulted in significant numbers of US military personnel returning from theaters of operation with lasting health issues including post-traumatic stress disorder (PTSD), depression, and other mental health morbidities and negative behaviors associated with varying war-related exposures [2,3,4,5,6,7,8,9,10,11,12,13,14,15,16,17]. The US Department of Veterans Affairs released a report in 2023 estimating that up to 15% of Veterans who supported operations in GWOT experience PTSD in a given year and many suffer from co-occurring conditions [18].

Addressing the long-term health impact of GWOT deployment has leveraged significant advances in mental health treatment and comprehensive Veteran support services over the past decade, with continued efforts on advancement in accessibility, continuity of care, and treatment. To this goal, there has been a focus on mitigating PTSD symptoms as well as lessening the overall health impacts stemming from the wars [19]. Acupuncture has been suggested as a treatment though the clinical effects have been unclear [20]. Researchers have conducted randomized controlled clinical trials (RCTs) highlighting the clinically efficacious impact of implementing acupuncture treatment for military Veterans with PTSD symptoms [21,22,23]. While our original study showed acupuncture was superior to sham, this analysis will go further to investigate independent variable contribution. Due to PTSD comorbidities and other associated factors, in this secondary analysis, the purpose was to prospectively investigate subgroups of baseline comorbidities, demographic characteristics, and PTSD symptom reduction in a controlled clinical trial of acupuncture treatment for combat-related PTSD in military Veterans.

## 2. Materials and Methods

### 2.1. Population and Data Sources

**Figure 1:**
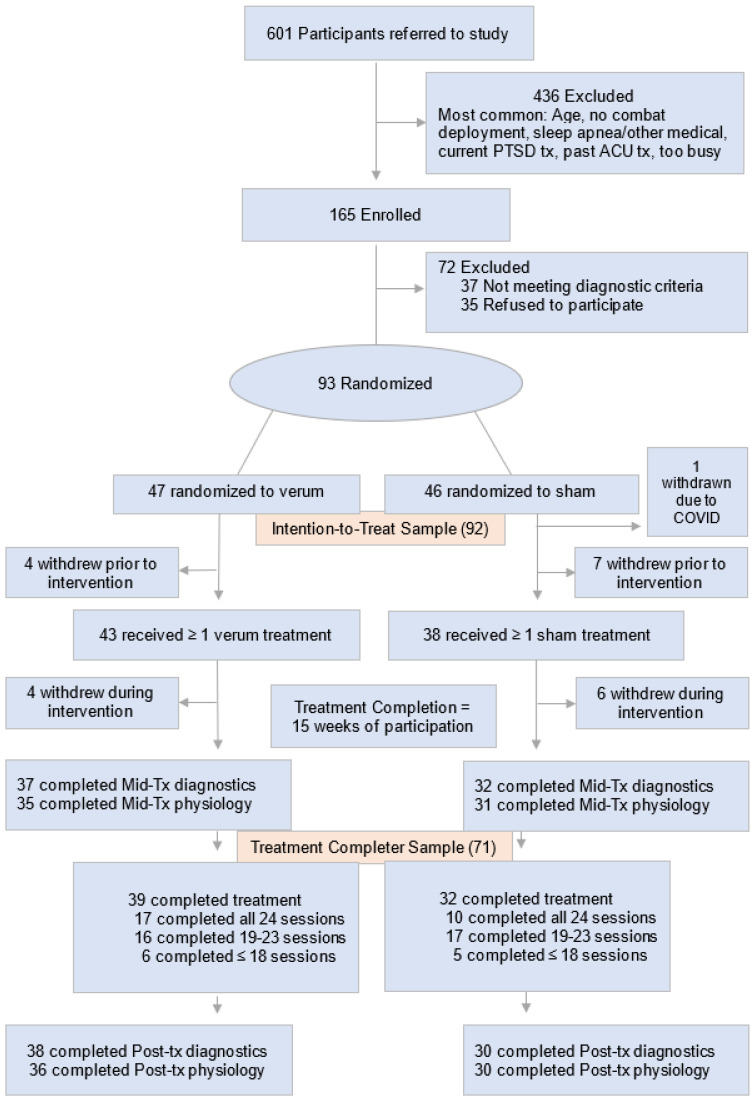
Participant Recruitment Flow Diagram. This figure was cited by Hsiao et al. [24].

This four-year, two-arm, parallel-group, placebo-controlled randomized clinical trial was conducted at the Long Beach VA Healthcare System (LBVA) [21,22]. The methods have been described in length elsewhere [21]. Briefly, the sample frame included treatment-seeking 18- to 55-year-old Veterans with chronic combat-related PTSD presenting for care at the LBVA or affiliated programs between April 2018 and May 2022. Seventy-one participants completed the intervention protocols from the ninety-three randomized (85 male and 8 females; mean [SD] age, 39.2 [8.5] years) of the n = 165 enrolled from the original n = 601 referred (Figure 1) [22,24]. Intention to treat and other investigations of differences in drop outs did not find discernible differences. This study was conducted in compliance with all applicable federal regulations governing the protection of human subjects in research and was approved by the Institutional Review Board and the Research and Development Committee at the Tibor Rubin VA Medical Center [21].

### 2.2. PTSD Assessment

Clinician Administered PTSD Scale-5 (CAPS-5), a 30-item, structured clinical interview designed to assess PTSD symptoms, was used to determine symptom severity. Interviews were conducted pre-treatment, at midpoint (mid-treatment), and post-treatment with an assessment window of ±3 days [21,25]. The frequency and intensity of symptoms were assessed and combined to generate an overall total symptom severity score, which also corresponds to a categorical diagnosis. CAPS-5 has demonstrated strong interrater reliability (κ = 0.78 to 1.00) and test–retest reliability (*κ* = 0.83) [25]. In this study, the CAPS-5 (past month version for baseline diagnosis, past week version at end-treatment) was administered by two psychologists trained in a validated telephone approach [21]. Twenty assessments were co-rated from audio-tape recordings with a symptom severity inter-rater reliability (Pearson’s *r*) of 0.91, *p* < 0.001.

For these analyses, the differential between pre and post was utilized to calculate mean differences and standard deviations. To identify a clinically relevant symptom reduction point for this investigation, the standard deviation of the change in CAPS-5 score from pre to post-assessment was calculated (SD = 12.3). Other studies have suggested a 15-point symptom reduction on the CAPS-5 to highlight a clinically significant or meaningful symptom improvement [26]. For this study, a 15-point reduction corresponds to roughly 1 SD and has been proposed as a minimally clinically important difference in prior CAPS-5 work. This cut point was used to create a dichotomous variable of clinical benefit for analyses.

### 2.3. Patient Characteristic Predictors

Patient characteristics were assessed at baseline with a provider-administered survey and included in these analyses were sex, age (continuous), country of origin (USA vs. outside of USA), education (college degree vs. other), work hours (continuous), marital status (married, single, or divorced/other), religion (some religion vs. none indicated), income ($50,000 or more annually vs. <$50,000 annually), employed (yes vs. no), combat exposure (categorized from 5 categories into moderate/moderate heavy/heavy, vs. light/moderate light), and deployment preparedness (low, moderate, or high).

### 2.4. Baseline Self-Reported Clinical Predictors

Exploring predictors of PTSD treatment response in an acupuncture versus sham RCT requires assessment of a constellation of patient characteristics including previously mentioned baseline demographics, but also goes further to include comorbidities, predisposition or survivor skills, functional capabilities, and current pain status. Poorer treatment responses in trauma-focused psychotherapy have reported factors in addition to demographics such as sleep, pain, poor quality of life, trauma-related cognitions, and depression, among others [27]. This exploratory analysis utilized many of these factors to determine which ones, in the context of the others, were most predictive in this small population of combat Veterans.

The Adverse Childhood Events Questionnaire (ACE) was originally developed to measure the impact of various forms of childhood abuse and neglect upon health and well-being among participants in the original 1998 Adverse Childhood Experiences study, conducted by CDC and Kaiser Permanente. It consists of 10 yes/no questions assessing adverse events before the age of 18, with higher scores indicating greater exposure to various forms of abuse, neglect, or household dysfunction. The ACE has demonstrated acceptable internal consistency (Cronbach’s alpha ~0.70–0.79) [28] and strong predictive validity for a number of long-term health outcomes, including PTSD [29,30,31,32,33].

The Dissociative Experiences Scale (DES-II) is a 28-item self-report questionnaire that assesses the frequency of dissociative experiences (amnesia, depersonalization, derealization, absorption, and identity alteration) on a 10-point Likert scale [34]. Total scores are calculated by averaging the scores of the 28 items, with a score of at least 30 suggesting high dissociative experiences. The scale has a high test–retest reliability of 0.78–0.93, an internal reliability of 0.93, and a convergent validity of 0.67 [35]. The instrument has been utilized in research studies on various psychiatric disorders including borderline personality disorder and PTSD [36,37].

The Hamilton Anxiety Rating Scale (HAM-A) was used to measure the severity of anxiety symptoms [38]. Participants were asked to rate 14 items on a Likert scale (0–4), where <17 indicates mild anxiety, 18–24 mild to moderate, 25–30 moderate to severe, and greater than 30 signifying severe anxiety.

The New Mexico Symptom Checklist—Somatic Scale (NMSCL-SOM) assesses physical symptoms that cause individuals distress through a 39-item questionnaire. Each item is rated on a 0–4 scale with 0 meaning the symptom is not experienced and 4 meaning the symptom is extremely bothersome. The NMSCL-SOM items are a part of the larger New Mexico Symptom Checklist-121 [39]. The original 121 item scale has been shown to be valid and reliable (*r* ≈ 0.80) in Vietnamese and Kurdish populations and been used to inform widely adopted refugee screening tools [40].

Beck’s Depression Inventory (BDI-II) is a widely used 21-item self-report questionnaire assessing severity of depressive symptoms with high internal consistency [41]. Scores range from 0 to 63, with higher scores indicating more severe depressive symptoms. The scores are typically categorized into ranges to indicate the level of depression: 0–13 for minimal depression, 14–19 for mild depression, 20–28 for moderate depression, and 29–63 for severe depression.

The McGill Pain Questionnaire (MPQ) is a multi-dimensional scale used widely to evaluate patients’ pain experiences across various conditions [42,43]. The measure uses descriptors that fall into four major categories: sensory, affective, evaluative, and supplementary. The rank for each descriptor is based on its position in the word set, the sum of which constitutes the pain rating index (PRI). The present pain index (PPI) is scored on a Likert scale, ranging from 0 (none) to 5 (excruciating). While there is little research on the predictive validity of the MPQ, there is evidence that the scale can differentiate between types of pain conditions, is reliable, and sensitive to change [44].

The Emotional Dysregulation Scale (EDS) is a 12-item self-report questionnaire designed to measure the severity of emotional dysregulation [45]. Three aspects of emotional dysregulation (cognitions, behaviors, and how a person subjectively experiences their emotions) are measured on a 7-point Likert scale, ranging from 1 (“Not true”) to 7 (“Very true”), with higher scores indicating greater levels of dysregulation. The EDS-short scale has demonstrated high internal consistency (Cronbach’s alpha of 0.93–0.95), good construct validity, and strong convergent validity with the measure’s longer version (r = 0.98).

The Veteran RAND 12 (VR12) is a 12-item self-report questionnaire for assessing health-related quality of life and disease burden [46]. The questionnaire queries several health domains, including “general health perceptions, physical functioning, role limitations due to physical and emotional problems, bodily pain, energy–fatigue, social functioning, and mental health” [46]. Response items are summarized into two scores—a Physical Component Summary (PCS) score and a Mental Component Summary (MCS) score. For all 12 questions, respondents can choose from a Likert scale to select their best response. Higher summary scores are interpreted as having less disease burden and better quality of life. The VR12 is a valid and reliable measure and has been utilized with Veterans since 1997 [47,48]. Internal consistency reliability (Cronbach *α*) is estimated to be at 0.90 for both PCS and MCS. PCS and MCS scores were also found to account for 92% of the reliable variance from previous VR versions.

Pittsburgh Sleep Quality Index (PSQI) is a self-rated questionnaire assessing sleep quality and disturbances over a 1-month time interval. The PSQI consists of 19 individual items that generate 7 component scores, whose sum yields a global score with a possible range of 0–21 and has been reported to be internally consistent and reliable [49].

The total Weekly Safety Survey (SAFE) score is scored from a three-item questionnaire made up of items derived from the Beck Depression Inventory (BDI-II) measure, specifically with items assessing suicidal thoughts and dying, irritability, and agitation [50]. Each item is rated on a scale of 0 to 3 with higher scores indicating greater symptom severity. While this composite of items has not been validated in the broader literature, the original measure (BDI-II) demonstrates excellent internal consistency (*α* ≈ 0.9–0.94) and test–retest reliability (*r* = 0.73–0.96), in addition to strong validity across clinical and cultural populations [41,50,51].

The Aggression Questionnaire is a 10-item subscale utilizing a 5-point Likert scale, scored from 1 (extremely uncharacteristic of me) to 5 (extremely characteristic of me) [52]. It is derived from Buss & Perry’s Aggression Questionnaire (1992), which has been used in prior research as a measure for trait aggressivity as a mediator of alcohol-related aggression [53], self-reported aggression following an 8-week Mindfulness-Based Resilience Training [54], and aggressive behavior among individuals with bipolar disorder [55]. Cronbach’s alpha for the original instrument was found to be 0.85, with alphas for the scale ranging from 0.72 to 0.85 [52].

The Credibility Rating Score (CRS) is calculated from the dual factor questionnaire developed utilizing a 1–9 Likert scale from 1 (not at all logical/useful/confident) to 9 (very logical/useful/confident) [56]. The credibility factor has high internal consistency and reliability as demonstrated by a Cronbach *α* between 0.81 and 0.86 and test–retest reliability of 0.75, respectively. The full scale is used widely in clinical research to measure treatment credibility and expectations for improvement, particularly in trials exploring digital interventions for psychiatric disorders like depression [57] and OCD [58], and acupuncture as a treatment for allergic rhinitis and chronic lower back pain (Sung et al., 2020) [59,60].

The Total Expectancy Score (ES) is a standardized total from the CRS using item 4 from set 1, and items 5 and 6 from set 2 which originated from the Credibility/Expectancy questionnaire (CEQ) [56]. Both items 4 and 6 are rated on a percentage scale of 0–100 by intervals of 10, and item 5 is rated on a Likert scale ranging from 1–9, with higher scores across all items indicating a greater expectancy for improvement in symptoms due to treatment. The CEQ has shown great internal consistency (α ≈ 0.90) and good test–retest reliability (r ≈ 0.82), and later validations showing a close range (α ≈ 0.75) across cultural contexts and patient populations [56,61,62].

### 2.5. Statistical Analyses

Descriptive analyses of demographic, clinical assessments, and intervention variables by the 15-point or more reduction in CAPS-5 status were completed. Univariate analyses, including Chi-square and *t*-tests were performed to assess the significance of unadjusted associations. A multivariable model was conducted to assess multicollinearity while simultaneously adjusting for all other covariates in the model. Hierarchical multivariable logistic regression models were then conducted to compare the predictive capabilities using the concordance statistic (c-statistic) to measure how well the models discriminated between the CAPS-5 15-point or more reduction and less than 15-point reduction at follow-up. The c-statistic ranges from 0.5 to 1.0 with interpretation of 1.0 being considered as perfect discrimination (0.7 as acceptable, 0.8 as strong, and 0.9 or above considered outstanding) [63]. These models allowed for further investigation of the adjusted odds as well as the Chronbach *α* for investigating how the set of items measure the same underlying construct (<0.5 = limited to not at all, 0.5–0.6 = poor, and 0.6–0.7 = questionable) [64]. The first model only included demographic characteristics while the second model only included baseline clinical assessments. A third model included all demographic and clinical variables, a fourth model only included the intervention variable, and the last model in the progression included all variables in a full model. The full model was run to calculate standardized coefficients providing for a rank order of the predictors of 15-point reduction, relevant to all other variables in the model. Lastly, a full multivariable backwards logistic regression model was conducted retaining all variables in the model at alpha < 0.10. Hosmer–Lemeshow goodness-of-fit test statistics are included. Statistical analyses were performed using SAS software (version 9.1.3, SAS Institute, Inc., Cary, NC, USA).

## 3. Results

Table 1 describes demographic, clinical, and intervention factors stratified by whether the participant had a 15-point or more reduction in CAPS-5 score at follow-up. The PCS from the VR12 and MPQ were the only two variables statistically associated with a 15-point reduction in CAPS-5, suggesting that those with better physical functioning (PCS) and those indicating less bodily pain (MPQ) were statistically more likely to have a 15-point reduction unadjusted for other factors. Though not statistically significant, Veterans who were 50 years or older, female, born outside the U.S., college educated, married, practicing some type of religion, earning >$50,000, not employed, reporting moderate to heavy combat exposures, or reporting high deployment preparedness were proportionally more likely to indicate a 15-point reduction based on a Chi-square test. Further, though not statistically significant, baseline health assessments indicated those reporting less impairment on the DES-II, BDI-II, EDS, PSQ, and Aggression Questionnaire and more impairment on the HAM-A, NMSCL-SOM, MCS, and credibility rating scale were more likely to have a 15-point reduction in CAPS-5 at follow-up.

There was notable multicollinearity found between work hours and employment (variance inflation >10.0). There was no additional discernible multicollinearity though variance inflations ranged between 3.0 and 3.7 for NMCSL-SOM, BDI-II, EDS, and MCS.

Table 2 presents the results of the hierarchical multivariable logistic modeling. The demographic variables alone provided a nearly acceptable prediction of 15-point reduction (c-statistic = 0.69) and indicated limited internal consistency (Cronbach *α* = 0.41). The baseline clinical assessments provided an acceptable prediction (c-statistic = 0.75) with limited internal consistency (Cronbach’s *α* = 0.51), suggesting assessment of a wider range of variability in the model. Together, the demographic variables along with the clinical baseline variables indicated strong prediction (c-statistic = 0.92) and the addition of the group intervention variable increased this slightly (c-statistic = 0.94) with limited internal consistency and Hosmer–Lemeshow goodness-of-fit statistic = 0.4673 indicating a good fit.

Table 3 provides understanding of the relative importance of a standardized coefficient in the rank order of the predictors of the outcome, relevant to all other variables in the model. The five most influential demographic variables included age, employment, income, deployment readiness, and sex. Age was the second most influential variable overall and the first most among demographic variables in predicting CAPS-5 reduction resulting in an increase of 1.32 in odds of reduction in symptoms for each 1-year increase in age after adjusting for all other variables in the model (95% CI = 1.03–1.70; *p*-value = 0.03). Employment, income, and deployment readiness were the second, third, and fourth most predictive demographic variables and exhibited an inverse relationship to CAPS-5 reduction, suggesting those who were employed, those with a higher income, and those who were more combat deployment ready were at a decrease in adjusted odds of symptom reduction when compared to those non-employed, those with a lower income, and those who were less combat prepared, respectively, after adjusting for all other variables in the model (these variables were not statistically significant). Sex was ranked as the 5th most influential demographic variable with females significantly more likely to have a 15-point symptom reduction when compared to males after adjusting for all other variables in the model (Adjusted OR = 149.05; 95% CI = 1.15–19,309.81; *p*-value = 0.04).

The five most influential baseline clinical assessment variables included PCS, EDS, MCS, MPQ, and BDI-II. PCS was the most influential variable in predicting CAPS-5 reduction with 1.30 odds of 15-point reduction in symptoms for each 1-point increase after adjusting for all other variables in the model (95% CI = 1.02–1.66; *p*-value = 0.03). Investigation of the Hosmer–Lemeshow goodness-of-fit statistic = 0.1591 indicated a good fit.

Table 4 presents the results of a multivariable backwards stepwise logistic regression using alpha = 0.10. Though the confidence interval was large and included 1, females may be more likely to have a 15-point CAPS-5 reduction at follow-up when compared to males (AOR = 14.7, 95% CI = 0.99 to 219.95) after controlling for other variables in the model. Participants treated with acupuncture were over four times more likely to have a 15-point CAPS-5 reduction at follow-up when compared to participants receiving sham (AOR = 4.17, 95% CI = 1.19 to 14.62) after controlling for other variables in the model. Those indicating better physical health at baseline were at 1.14 times the odds of a 15-point CAPS-5 reduction at follow-up for each 1 unit increase in physical functioning score when compared to participants reporting lower PCS scores at baseline (95% CI = 1.03 to 1.25) after controlling for other variables in the model. Investigation of the Hosmer–Lemeshow goodness-of-fit statistic = 0.6745 indicated a good fit.

## 4. Discussion

Identifying markers that may symbiotically contribute to the therapeutic benefits of treatments or procedures is critical to helping minimize the potentially long-lasting burden of PTSD. This study utilized an RCT to investigate a pre/post acupuncture intervention resulting in a clinically relevant 15-point reduction in CAPS-5 symptoms. Though exploratory in nature, the consistent findings of less pain and better physical functioning at baseline being predictive of PTSD symptom reduction offers a possible synergy that warrants further exploration. A previous report indicated that low physical and mental functioning prior to combat is associated with PTSD symptoms after deployment [65]. Further, it has been reported that improvements in pain and physical functioning may provide parallel benefits for depressive and anxiety symptoms [66,67]. Recent reports from a PTSD clinical trial suggested that increased baseline pain severity predicted a slower response to treatment [68], whereas a report of cognitive processing therapy concluded that physical functioning impacted PTSD symptom improvement [69]. These studies provide a clear interrelationship between pain, physical health, and PTSD symptoms though have not established how physical health at baseline may benefit symptom reduction over time. This study offered another view of this interrelationship.

Our finding of those not employed while also reporting earning above $50,000 per year aligns with a retired population and is consistent with a recent report suggesting nearly 50% of military members separating from service are routinely retired with only 17% unemployed [70]. Further, Veterans who are not employed, retired, and report higher income might have more time and financial resources to allow them to be more adherent to acupuncture treatment and “lean in” to treatment, which may differ from those not employed though not retired and report lower incomes.

Though the point estimate suggested markedly higher odds among women, the very wide confidence interval may indicate uncertainty. Still, the hypothesis generating the finding that females were at increased adjusted odds of CAPS-5 symptom reduction is interesting and requires further research. Females in the military experience many of the same combat and deployment experiences as males resulting in increased mental health issues [71]. In a recent report, females were at increased odds for additional sexual stressors their male counterparts may not experience leading to additional mental health challenges among females [72].

The more than four-fold adjusted odds of symptom reduction found in those receiving the acupuncture intervention versus sham is not surprising based on the recent published report focused on the main objectives of the RCT finding acupuncture to be superior to sham acupuncture on symptom reduction. What may be surprising is that the authors did not explore the predictive nature of the acupuncture intervention independently of the many other clinical and demographic variables. This may have clinical implications, since here, we find both an independent and interactional relationship of acupuncture to other demographic and clinical variables on symptom reduction, highlighting the potential importance of patient selection for achieving a good outcome. If these relationships are prospectively determined, then efficient treatment with cost savings might ensue.

Limitations exist in these analyses. The study population is a relatively small and self-selected VA treatment seeking study population of combat Veterans who are mostly male, with a limited age range who may not be generalizable to all military members. This has clear implications in model building and the number of variables included as does the loss to follow-up which may bias the results if informative. Self-reported assessment of exposure and clinical outcomes represents an estimate of the true prevalence and has inherent limitations that may lead to information bias in these analyses. Dichotomizing a continuous CAPS variable was intended to enhance and simplify predictive information though there may be some loss of information stemming from the CAPS measurement. Some of the confidence intervals were large, indicating variability that may only offer a trend and help to generate future hypotheses. Factors such as baseline expectancy and Credibility Rating Score, among others, did not emerge as strong predictors in these analyses and could be due to other stronger predictors, make-up of sample, or other limitations. The relatively low Chronbach’s Alpha across the total scores of the many measures coupled with a low variance inflation suggested these total measures were assessing different variance.

There are numerous unique strengths in this study including the prospective RCT design to assess baseline and post intervention effect, many demographic and military variables, and multiple standardized instruments for clinical assessment. While self-reported data may have limitations, these data cannot be assessed elsewhere and present an extensive clinical and demographic view of the patient.

In summary, this prospective study exploring predictors of PTSD symptom reduction adds to a growing body of evidence demonstrating the complex relationship between physical functioning, pain, demographic factors, PTSD, and potential treatments. The modest sample size coupled with self-reported measures impact the generalizability of findings; however, the prospective randomized controlled design and comprehensive assessment of demographic and clinical data using validated instruments strengthen the relevance of this work and allow for prospective exploration of predictive impact of these many variables. This study highlights the potential for integrative therapies and clinical reduction in comorbidities to complement existing treatment approaches to achieve PTSD symptom reduction. Further, the original paper indicated a strong effect of acupuncture over sham in the overall study [22]. Further research should be considered focused on groups with clinical and demographic characteristics identified in the current study to investigate the heightened effect of acupuncture treatment for meaningful PTSD symptom reduction in target populations.

## 5. Implications and Contribution

This study identifies a set of patient characteristics, both clinical and demographic, that may allow for a target group of PTSD patients for whom acupuncture treatment may be maximized.

## Figures and Tables

**Table 1:** Characteristics of 71 patients indicating 15 or more point CAPS-5 reduction.

			Fifteen-Point Reduction			
	Population N = 71		No n = 37		Yes n = 34	
	N	(%)	N	(%)	N	(%)
Age Group, in years						
25–29	9	(12.7)	6	(16.2)	3	(8.8)
30–34	15	(21.1)	5	(13.5)	10	(29.4)
35–39	17	(23.9)	11	(29.7)	6	(17.6)
40–44	7	(9.9)	5	(13.5)	2	(5.9)
45–49	12	(16.9)	7	(18.9)	5	(14.7)
50+	11	(15.5)	3	(8.1)	8	(23.5)
Gender						
Male	65	(91.5)	35	(94.6)	30	(88.2)
Female	6	(8.5)	2	(5.4)	4	(11.8)
Country of Birth						
USA	54	(76.1)	30	(81.1)	24	(70.6)
Other	17	(23.9)	7	(18.9)	10	(29.4)
Education Level						
College	26	(36.6)	12	(32.4)	14	(41.2)
HS/GED/Some College	45	(63.4)	25	(67.6)	20	(58.8)
Marital Status						
Married	39	(54.9)	19	(51.4)	20	(58.8)
Single	14	(19.7)	7	(18.9)	7	(20.6)
Divorced/Other	18	(25.4)	11	(29.7)	7	(20.6)
Religion						
Some Type Religion	57	(80.3)	28	(75.7)	29	(85.3)
No Religion Indicated	14	(19.7)	9	(24.3)	5	(14.7)
Annual Income						
<$49,999 or Missing	36	(50.7)	20	(54.1)	16	(47.1)
>$50,000	35	(49.3)	17	(45.9)	18	(52.9)
Employed						
No	42	(59.2)	20	(54.1)	22	(64.7)
Yes	29	(40.8)	17	(45.9)	12	(35.3)
Race Ethnicity						
Other	11	(15.5)	2	(5.4)	9	(26.5)
Hispanic/Latino	31	(43.7)	20	(54.1)	11	(32.4)
Non-Hispanic White	15	(21.1)	7	(18.9)	8	(23.5)
Non-Hispanic Asian	14	(19.7)	8	(21.6)	6	(17.6)
Combat Exposure						
Light, Moderate Light	15	(21.1)	10	(27.0)	5	(14.7)
Moderate, Heavy	56	(78.9)	27	(73.0)	29	(85.3)
Deployment Preparedness						
Low Preparedness	6	(8.5)	3	(8.1)	3	(8.8)
Moderate Preparedness	33	(46.5)	19	(51.4)	14	(41.2)
High Preparedness	32	(45.1)	15	(40.5)	17	(50.0)

	Mean (SD)		Mean (SD)		Mean (SD)	
Age	39.5 (8.7)		38.7 (7.8)		40.5 (9.6)	
**Baseline Clinical Assessments**						
Adverse Childhood Experience (ACE) Total	3.5 (2.7)		3.4 (2.6)		3.6 (2.7)	
Dissociative Experiences Scale (DES-II) Total	29.6 (17.8)		30.7 (18.7)		28.5 (17.1)	
Hamilton Anxiety Rating Scale (HAM-A) Total	33.1 (9.6)		34.8 (9.3)		31.3 (9.7)	
New Mexico Symptom Checklist—Somatic Scale (NMSCL-SOM)	55.6 (26.2)		59.8 (24.7)		51.1 (27.4)	
Beck Depression Inventory (BDI-II)	31.0 (11.3)		33.1 (11.9)		28.8 (10.4)	
McGill Pain Questionnaire (MPQ)	30.4 (14.7)		**34.2 (15.1)**		**26.2 (13.1)**	
Emotion Dysregulation Scale (EDS)	58.7 (16.9)		61.1 (16.4)		56.1 (17.4)	
VR-12 is the Veterans RAND 12-item Health Survey Mental Component Summary (MCS)	31.5 (9.3)		30.2 (9.2)		32.9 (9.4)	
VR-12 is the Veterans RAND 12-item Health Survey Physical Component Summary (PCS)	38.9 (10.7)		**36.0 (9.4)**		**42.1 (11.2)**	
Pittsburg Sleep Quality Index (PSQ)	14.0 (3.5)		14.7 (3.1)		13.2 (3.9)	
SAFE	2.4 (2.0)		2.5 (1.9)		2.4 (2.1)	
Aggression Questionnaire	26.5 (8.0)		27.4 (8.6)		25.5 (7.3)	
Credibility Rating Scale (CRS)	0.27 (2.3)		−0.1 (2.4)		0.7 (2.0)	
Expectancy Score (ES)	0.02 (2.6)		−0.4 (2.6)		0.5 (2.6)	

Bold text indicates statistically significant differences (*p*-value < 0.05) between those with a 15-point reduction in pre/post CAPS-5 scores and baseline demographic or clinical variables.

**Table 2:** Prediction of CAPS-5 15-point reduction using hierarchical characteristic groupings (N = 71).

Grouping of Variables	c-Statistic *	Standardized Chronbach’s Alpha **
**Demographics:** sex, age, country, education, weekly work hours, marital status, religion, income, employment, combat exposure, deploy preparedness	0.69	0.41
**Baseline Clinical Assessments:** Adverse Childhood Experience (ACE), Dissociative Experiences Scale (DES-II), Hamilton Anxiety Rating Scale (HAM-A), New Mexico Symptom Checklist—Somatic Scale (NMCSL-SOM), Beck Depression Inventory (BDI-II), McGill Pain Questionnaire (MPQ), Emotion Dysregulation Scale (EDS), VR-12 is the Veterans RAND 12-item Health Survey Mental Component Summary (MCS), VR-12 is the Veterans RAND 12-item Health Survey Physical Component Summary (PCS), Pittsburg Sleep Quality Index (PSQ), **SAFE**, Aggression Questionnaire, Credibility Rating Scale (CRS), Expectancy Score (ES)	0.75	0.51
**All Demographics and Clinical Baselines**	0.92	0.32
**Intervention Group Only**: Acupuncture or Placebo	0.68	
**All Variables**	0.94	0.31

* C-statistic measures how well a model discriminates between 15-point reduction and not; 0.5 is chance, 0.7–0.8 is acceptable, 0.8–0.9 is excellent, >0.9 is near perfect. ** Cronbach’s Alpha calculates how well a set of items measure the same underlying construct; <0.5 is limited to not at all, 0.5–0.6 is poor, 0.6–0.7 is questionable.

**Table 3:** Full multivariable logistic regression modelling the odds of 15-point CAPS-5 reduction in pre/post assessment.

Variable	Standardized Coefficient	Adjusted Odds Ratio *	Lower 95% Confidence Limit	Upper 95% Confidence Limit	*p*-Value **
VR-12 is the Veterans RAND 12-item Health Survey Physical Component Summary (PCS)	1.540	1.30	1.02	1.66	**0.03**
Age, in years	1.340	1.32	1.03	1.70	**0.03**
Emotion Dysregulation Scale (EDS)	−1.248	0.87	0.75	1.02	0.09
Employment (yes vs. no)	−1.137	0.02	0.00	1.01	**0.05**
VR-12 is the Veterans RAND 12-item Health Survey Mental Component Summary (MCS)	−1.028	0.82	0.64	1.04	0.10
Income ($50,000 or more vs. <$50,000)	−0.970	0.03	0.00	1.12	0.06
Deployment Prepared (high vs. low)	−0.881	0.04	0.00	4.97	0.19
Sex (female vs. male)	0.778	149.05	1.15	19,309.81	**0.04**
Marital Status (divorced/other vs. married)	−0.768	0.04	0.00	1.62	0.09
McGill Pain Questionnaire (MPQ)	−0.672	0.92	0.83	1.02	0.11
Intervention Group (acupuncture vs. sham)	0.656	10.72	0.99	116.62	**0.05**
Beck Depression Inventory (BDI-II)	−0.619	0.91	0.77	1.06	0.22
Marital Status (single vs. married)	−0.614	0.06	0.00	2.38	0.14
Religion Status (some type vs. none)	0.606	15.29	0.62	374.44	0.09
Credibility Rating Scale (CRS)	0.552	1.56	0.83	2.91	0.17
Combat Exposure (moderate/high vs. low)	0.534	10.43	0.58	188.90	0.11
Deployment Prepared (moderate vs. low)	−0.502	0.16	0.00	10.13	0.39
Pittsburg Sleep Quality Index (PSQ)	−0.353	0.83	0.59	1.19	0.32
Expectancy Score (ES)	0.349	1.27	0.77	2.08	0.34
SAFE	−0.311	0.75	0.39	1.47	0.41
Dissociative Experiences Scale (DES-II)	0.307	1.03	0.95	1.11	0.43
New Mexico Symptom Checklist—Somatic Scale (NMCSL-SOM)	−0.296	0.98	0.91	1.05	0.57
Aggression Questionnaire	0.161	1.04	0.84	1.28	0.73
Hamilton Anxiety Rating Scale (HAM-A)	−0.063	0.99	0.87	1.13	0.86
Country of Birth (USA vs. other)	−0.048	0.81	0.07	9.62	0.87
Education (college vs. less than college)	0.038	1.15	0.12	11.38	0.90
Adverse Childhood Experience (ACE)	0.035	1.02	0.69	1.52	0.91

* Adjusted odds ratio adjusting for all other variables in the model after backwards stepwise regression and elimination of variables from the full model in Table 3. ** *p*-values based on the Wald Chi-square test.

**Table 4:** Backwards stepwise multivariable logistic regression modelling the odds of 15-point CAPS-5 reduction in pre/post assessment with replacement using alpha = 0.10 for final inclusion.

Variable	Adjusted Odds Ratio *	Lower 95% Confidence Limit	Upper 95% Confidence Limit	*p*-Value **
McGill Pain Questionnaire (MPQ)	0.95	0.90	1.00	0.08
Age, in years	1.12	1.01	1.24	0.03
Employment (yes vs. no)	0.06	0.01	0.45	0.01
Intervention Group (acupuncture vs. sham)	4.17	1.19	14.62	0.03
Emotional Dysregulation Scale (EDS)	0.95	0.91	1.00	0.05
VR-12 is the Veterans RAND 12-item Health Survey Physical Component Summary (PCS)	1.14	1.03	1.25	0.01
Religion Status (some type vs. none)	5.60	0.74	42.33	0.10
Sex (female vs. male)	14.72	0.99	219.95	0.05

* Adjusted odds ratio adjusting for all other variables in the model after backwards stepwise regression and elimination of variables from the full model in Table 3. ** *p*-values based on the Wald Chi-square test.

## Data Availability

The original contributions presented in the study are included in the article, further inquiries can be directed to the corresponding authors.

## Abbreviations

Post-traumatic Stress Disorder (PTSD); Clinician Administered PTSD Scale-5 (CAPS-5); Adverse Childhood Events Questionnaire (ACE); Dissociative Experiences Scale (DES-II); Hamilton Anxiety Rating Scale (HAM-A); New Mexico Symptom Checklist—Somatic Scale (NMSCL-SOM); Beck’s Depression Inventory (BDI-II); McGill Pain Questionnaire (MPQ); Emotional Dysregulation Scale (EDS); Veteran RAND 12 (VR12); Pittsburgh Sleep Quality Index (PSQI); Weekly Safety Survey (SAFE); Credibility Rating Score (CRS); Expectancy Score (ES).
